# Pregestational Exposure to *T. gondii* Produces Maternal Antibodies That Recognize Fetal Brain Mimotopes and Induces Neurochemical and Behavioral Dysfunction in the Offspring

**DOI:** 10.3390/cells11233819

**Published:** 2022-11-29

**Authors:** Eunice Romero Núñez, Tonali Blanco Ayala, Gustavo Ignacio Vázquez Cervantes, Gabriel Roldán-Roldán, Dinora Fabiola González Esquivel, Saé Muñiz-Hernández, Alelí Salazar, Maricela Méndez Armenta, Saúl Gómez-Manzo, Hugo González-Conchillos, Angélica Luna-Nophal, Alma Patrica Acosta Ramírez, Benjamín Pineda, Anabel Jiménez-Anguiano, Verónica Pérez de la Cruz

**Affiliations:** 1 Posgrado en Ciencias Biológicas y de la Salud, DCBS, Universidad Autónoma Metropolitana-Iztapalapa, Ciudad de Mexico 09340, Mexico; 2 Neurobiochemistry and Behavior Laboratory, National Institute of Neurology and Neurosurgery “Manuel Velasco Suárez”, Mexico City 14269, Mexico; 3 Department of Physiology, Faculty of Medicine, National Autonomous University of México, Mexico City 04510, Mexico; 4 Laboratorio de Oncología Experimental, Subdirección de Investigación Básica, Instituto Nacional de Cancerología, Secretaria de Salud, Ciudad de México 14080, Mexico; 5 Neuroimmunology Department, National Institute of Neurology and Neurosurgery “Manuel Velasco Suárez”, Mexico City 14269, Mexico; 6 Experimental Neuropathology Laboratory, National Institute of Neurology and Neurosurgery “Manuel Velasco Suárez”, Mexico City 14269, Mexico; 7 Laboratorio de Bioquímica Genética, Instituto Nacional de Pediatría, Secretaría de Salud, México City 04530, Mexico; 8 Department of Infectomics and Molecular Pathogenesis, Center for Research and Advanced Studies (Cinvestav), México City 07360, Mexico; 9 Neurosciences Area, Biology of the Reproduction Department, Universidad Autónoma Metropolitana, Ciudad de México 09340, Mexico

**Keywords:** *T. gondii*, schizophrenia, cognitive impairment, molecular mimicry, social alterations

## Abstract

The activation of the maternal immune system by a prenatal infection is considered a risk factor for developing psychiatric disorders in the offspring. *Toxoplasma gondii* is one of the pathogenic infections associated with schizophrenia. Recent studies have shown an association between high levels of IgG anti-*T. gondii* from mothers and their neonates, with a higher risk of developing schizophrenia. The absence of the parasite and the levels of IgGs found in the early stages of life suggest a transplacental transfer of the anti-*T. gondii* IgG antibodies, which could bind fetal brain structures by molecular mimicry and induce alterations in neurodevelopment. This study aimed to determine the maternal pathogenic antibodies formation that led to behavioral impairment on the progeny of rats immunized with *T. gondii*. Female rats were immunized prior to gestation with *T. gondii* lysate (3 times/once per week). The anti-*T. gondii* IgG levels were determined in the serum of pregestational exposed females’ previous mating. After this, locomotor activity, cognitive and social tests were performed. Cortical neurotransmitter levels for dopamine and glutamate were evaluated at 60 PND in the progeny of rats immunized before gestation (Pregestational group). The maternal pathogenic antibodies were evidenced by their binding to fetal brain mimotopes in the Pregestational group and the reactivity of the serum containing anti-*T. gondii* IgG was tested in control fetal brains (non-immunized). These results showed that the Pregestational group presented impairment in short and long-term memory, hypoactivity and alteration in social behavior, which was also associated with a decrease in cortical glutamate and dopamine levels. We also found the IgG antibodies bound to brain mimotopes in fetuses from females immunized with *T. gondii*, as well as observing a strong reactivity of the serum females immunized for fetal brain structures of fetuses from unimmunized mothers. Our results suggest that the exposure to *T. gondii* before gestation produced maternal pathogenic antibodies that can recognize fetal brain mimotopes and lead to neurochemical and behavioral alterations in the offspring.

## 1. Introduction

*Toxoplasma gondii* is a parasite that can infect practically all nucleated cell types of warm-blooded animals, including humans. In healthy individuals, *T. gondii* infection is rapidly controlled and only results in mild illness. However, in experimental rodent models, chronic infection within the central nervous system (CNS) has been shown to exert a myriad of central alterations including variation of the neurotransmitters dopamine, γ-aminobutyric acid, and glutamate, loss of synapse formation in the cortex and hippocampus, reduction in dendritic spines, loss of fiber density in the cortex and altered synaptic protein composition [1]. These observations together with epidemiologic evidence in humans showing an association of maternal infection and immune activation, with the pathogenesis of neuropsychiatric disorders such as schizophrenia, raise the question of the mechanisms involved in triggering brain and behavioral alterations in the offspring. In this line, the neurodevelopmental theory of schizophrenia suggests that an event during the gestational period or early life negatively affects brain development by inducing changes in the brain structure, function, and activity, which later can lead to cognitive and psychosocial dysfunction [2,3]. Normally, the neurodevelopment process includes brain cell proliferation and migration, myelinization as well as the formation of neural circuits [4], so when some of these processes are disrupted it may result in long-term alterations in the adulthood. In support of the neurodevelopmental theory of schizophrenia studies of human subjects have showed solid evidence of abnormal brain structure, reduced neuropil density, impaired neuroplasticity, and abnormal neural circuits in patients with this schizophrenia [5,6,7]. The maternal immune activation (MIA) induced by infections such as influenza, rubella, herpes, polio, and toxoplasmosis, is one of those abnormal events occurring during neurodevelopment that are associated with the risk of schizophrenia and other psychiatric diseases [8,9,10,11]. In addition, preclinical studies support the idea that MIA induced by prenatal infections, rather than the direct action of infectious agents, is the mechanism that alters normal brain development increasing the risk of schizophrenia and other neuropsychiatric disorders [12,13]. Previous studies in rodents showed altered cytokine expression in the placenta, amniotic fluid and fetal brain after MIA induced by lipopolysaccharide (LPS) administration, suggesting the possibility that pro-inflammatory cytokines impair brain development leading to behavioral and cognitive disturbances such as those observed in schizophrenia [12,13,14,15,16,17,18,19].

Additionally, in the specific case of *T. gondii* infection, a study from serological biobanks demonstrates an association between elevated maternal IgG antibody to *Toxoplasma* and the risk of schizophrenia in adult offspring. In contrast, no subjects had elevated IgM titers excluding an active *T. gondii* infection and indicating the maternal origin of these immunoglobulins [10]. Furthermore, a birth cohort study showed that increased anti-*T. gondii* IgG levels in filter paper blood from newborns was associated with an increased risk of schizophrenia [20]. It is worth mentioning that elevated anti-*T. gondii* IgG antibodies were observed in the absence of an active infection. These studies suggest that the maternal anti-*T. gondii* IgG antibodies are transferred to the fetus during pregnancy and raise the question: what are the potential effects of persistently elevated maternal anti-*T. gondii* IgG antibodies make on the fetal brain? In similar approaches several groups have experimentally demonstrated that during gestation, IgG antibodies from mothers of children with autism spectrum disorders or mothers with lupus can reach the brain and thereby shown an association with behavioral alterations in the progeny [21,22,23,24]. In view of this background, it is feasible that early disruption of brain development by anti-*T. gondii* IgG may involve molecular mimicry with brain proteins that can alter the trajectory of brain development and physiology throughout adulthood. Thus, this study is focused on elucidating whether pregestational exposure to *T. gondii* induces maternal pathogenic antibodies that can recognize and bind to fetal brain mimotopes and lead to neurochemical, cognitive and social alterations in the offspring.

## 2. Materials and Methods

### 2.1. Animals

Male BALB/c mice were purchased from Unidad de Producción y Experimentación de animales de Laboratorio (UPEAL-CINVESTAV, Ciudad de México, México,) and Wistar rats were obtained from the animal house of the National Institute of Neurology and Neurosurgery (México City, México). These were used for this study. The animals were housed in acrylic cages under identical environmental conditions, i.e., temperature (25 ± 3 °C), humidity (50 ± 10%) and lighting (12 h light/dark cycles). The animals were provided with standard commercial rodent diet and water *ad libitum*. All experiments with animals were carried out according to the National Institutes of Health Guide for the Care and Use of Laboratory Animals, and the local guidelines on the ethical use of animals from the Health Ministry of Mexico. All efforts were made to minimize animal suffering during the study.

### 2.2. Materials

Reagents used in the purification of *T. gondii* tachyzoites were purchased from J.T. Baker Co. unless otherwise noted. Potassium phosphate, sodium octyl sulphate, EDTA, methanol, perchloric acid, sodium metabisulfite, tetrahydrofuran, o-phthalaldehyde (OPA), 2-mercaptoethanol and glacial acetic acid were obtained from Sigma Aldrich Company (St. Louis, MO, USA). Anti-rat IgG antibody coupled to Cy5 was obtained from Abcam, USA. All other chemicals were of the highest commercially available purity. Solutions were prepared using deionized water obtained from a Milli-RQ (Millipore, Burlington, MA, USA) purifier system. 

### 2.3. Toxoplasma Gondii Lysate

The *T. gondii* tachyzoites (RH strain) were harvested from the peritoneal cavity with 1X PBS (138 mM NaCl, 1.1 mM K_2_PO_4_, 0.1 mM Na_2_HPO_4_, and 2.7 mM KCl, pH 7.2) from previously inoculated BALB/c mice. The tachyzoites were washed 3 times with PBS, filtered through polycarbonate membrane filters (5 µm pore size) and centrifuged at 1500 rpm for 10 min. After this, the sediment containing pure *T. gondii* tachyzoites was resuspended in PBS. The total lysate of tachyzoites was obtained by 3 cycles of freezing in liquid nitrogen alternated with thawing in a 37 °C water bath (3 min each step), followed by lysate centrifugation at 3000× *g* for 15 min at 4 °C [25]. The protein quantification was done on a Nanodrop 2000 spectrophotometer. To validate the integrity of the antigens, a 7.5% polyacrylamide gel electrophoresis (SDS-PAGE) was performed, and the lysate was stored at −80 °C until used.

### 2.4. Rat Immunization

Female adult rats (2–3 months old) were separated into three groups (n = 8 each): (1) control group, rats that received PBS; (2) rats immunized with the *T. gondii* lysate prior to gestation (Pregestational); and (3) rats inoculated with *T. gondii lysate* at day 18 of gestation (Gestational). The control group received three subcutaneous injections with PBS before being pregnant, one injection per week. The pregestational group was injected subcutaneously with 100 µg of *T. gondii* lysate (~5 × 10^6^ parasites), once weekly for three weeks. The Gestational group received one subcutaneous injection of *T. gondii* lysate on gestational day 18.

### 2.5. Anti-T. gondii IgG Seropositivity

One week after the last immunization and before the gestational period, of the anti-*T. gondii* IgG titers were determined on the control group as well as on the Pregestational group. Serums were diluted 1:3 and analyzed using the Rat Toxoplasmosis (TOXO) Antibody (IgG) ELISA kit (NBP2-60168, Novus Biologicals, Centennial, CO, USA). Briefly, according to the manufacturer protocol, samples were pipetted into wells mixed with Horseradish Peroxidase (HRP)-conjugated anti-rat IgG in the microtiter plate precoated with antigen (TOXO). Any antibodies specific for the antigen present bound to the precoated antigen, a substrate solution was added, and color developed in proportion to the amount of rat anti-toxoplasmosis IgG bound. The intensity of the color was measured in a Synergy™ HTX multi-mode microplate reader (Biotek Instruments) at a wavelength of 450 nm. For calculating the valence of rat anti-toxoplasmosis (TOXO) antibody (IgG), the optical density (OD) of the sample was compared with the negative control: OD sample/OD negative ≥ 2.1: Positive and OD sample/OD negative < 2.1: Negative. Samples were measured for duplicate. 

### 2.6. Behavioral and Cognitive Test

#### 2.6.1. Locomotor Activity

Locomotor activity of the progeny from the three experimental groups was assessed using an Opto Varimex 4 system (Columbus Instruments, Columbus, OH, USA). The animals were first habituated to the system. After this, the locomotor activity was evaluated by placing each animal in the center of the cage and allowing them to explore the environment for 300 s. The cage was cleaned with alcohol (70%) between each experimental animal. The results are expressed as the total distance traveled (cm) and the vertical and horizontal movement counts recorded in 300 s.

#### 2.6.2. Novel Object Recognition (NOR) Test

Briefly, experimental rats were allowed to habituate to the box for 10 min. Then, the training phase started, in which two similar objects (A and A’) were placed diagonally opposite to each other and equidistant to the center of the box, and rats were allowed to explore the objects for 5 min. After one-hour, short term memory was evaluated, in this case by the rat being introduced again into the box and explored the objects for 5 min, but this time one of the familiar objects was changed for a novel object B. Then, long term memory was evaluated 24 h later. Rats were placed into the same box, but this time object B was removed, an object C was introduced, and the rat explored the objects for 5 min. Time spent by the rat sniffing and exploring each object was scored. The recognition index was calculated as time exploring novel object/time exploring both objects × 100.

#### 2.6.3. Crawley’s Test

Crawley’s test evaluates sociability and preference for social novelty. Briefly, the test is carried out in a rectangular box that contains three identical chambers. Each chamber measures 21 × 45 × 35 cm. The central chamber allows free access to the side compartments, in which two identical containers are placed. First, rats were habituated to the empty box for 10 min, and then held in the center chamber using the sliding doors. On the first session the experimental animal had free access to interact with another rat of the same age and sex locked in the container or with an empty one. In the second session, which is performed 24 h later, the experimental animal has free access to a familiar or an unfamiliar rat kept in the container. Each session has a 5 min duration and was video recorded for offline behavioral analysis [26,27]. The results are shown as the mean time spent in each chamber during each session.

### 2.7. Dopamine and Glutamate Determination

Brain tissue was rapidly collected after behavioral examination, and the cortex was dissected on ice. Dopamine (DA) was measured by HPLC analysis with electrochemical detection (LC-4C detector, BAS, West Lafayatte, IN, USA). Briefly, cortical tissue was homogenized in an antioxidant solution (0.4 N perchloric acid and 5.3 mM sodium metabisulfite, 10 µL/mg of tissue) and then centrifuged (10,000 rpm × 15 min); 100 µL of the supernatant were injected directly onto a 3 µm Adsorbosphere catecholamines column (4.6 × 100 mm, Alltech). Samples were eluted using a mobile phase (pH = 3.1) consisting of 23 mM potassium phosphate (KH_2_PO_4_), 2 mM sodium octyl sulphate, 0.55 mM EDTA and 15.5% methanol at a flow rate of 1 mL/min and oxidation potential of: +0.8 V. The retention time for DA was ~6 min.

Glutamate was measured by HPLC with fluorescence detection (S200a fluorescence detector; Perkin-Elmer, Waltham, MA, USA). Cortical tissue was homogenized in 40 volumes of 85% methanol/15% deionized water, centrifuged 4000× *g* × 10 min and further diluted 1:100 in MeOH/H_2_0. The supernatant was derivatized with o-phthalaldehyde (OPA)/2-mercaptoethanol. Fifty µL of derivatized samples were injected to a 3 µm C18 reverse phase column (ZORBAX Eclipse XDB 5 µm, 4.6 × 150 mm; Agilent, Santa Clara, CA, USA) and eluted by gradient using a mobile phase consisting of 2.4 mL/L glacial acetic acid and 1.5% *v*/*v* tetrahydrofuran (pH = 5.9). Glutamate was detected fluorometrically (excitation wavelength: 390 nm; emission wavelength: 460 nm) with a retention time of ~5.2 min.

### 2.8. Maternal Pathogenic Antibodies Identification by Immunofluorescence 

On gestational day 19, pregnant females from the experimental groups were sacrificed by a pentobarbital overdose and perfused transcardiacally with chilled 10% *w*/*v* buffered formalin solution. Fetal brains were obtained immediately after perfusion and fixed in 4% paraformaldehyde for 24 h at 4 °C under constant stirring. Subsequently, fetal brains were dehydrated and included in paraffin. Brain sections (5 μm thickness) were cut with a microtome (Microm, Waltham, MA, USA) and used for immunofluorescence assay. 

To evaluate the maternal pathogenic antibodies and their cross-reaction with fetal brain proteins, two strategies were followed: (1) anti-*T. gondii* IgG binding to brain structures was looked in histological sections of fetus brains from the Pregestational group and (2) the reactivity between the serum of females immunized prior gestation and the fetal brain structures of the offspring from non-immunized mothers. Briefly, one hemisphere of the fetal brain slices from the control and Pregestational group was blocked with 10% goat serum plus 0.1% Tween 20 in PBS for 30 min and incubated with anti-rat IgG-Cy5 (10 µg/mL diluted on 1% of BSA in PBS) for 30 min in the darkness. Then, the slices were washed two times with 1% BSA diluted in PBS and finally mounted with Vectashield/DAPI (Vector laboratories). For the second strategy, one hemisphere from the control fetal brains (mother non-immunized) was blocked with 10% goat serum plus 0.1% Tween 20 in PBS for 30 min and incubated with the maternal serum from control and immunized-prior-to-gestation mothers at room temperature for 30 min. Then, the sample was carefully washed with 0.1% of BSA diluted in PBS and incubated with a secondary anti-rat IgG antibody coupled to Cy5 fluorophore (10 µg/mL diluted on 1% of BSA in PBS) for 30 min in darkness. Finally, the brain slices were mounted with Vectashield/DAPI (Vector laboratories).

### 2.9. Protein Match between T. gondii and CNS

The three-dimensional structures of the GABR2 and MIC6 proteins were downloaded from the Uniprot platform. The structural conformation and pairwise alignment were carried out with the Match-maker extension of the UCSF Chimera program.

### 2.10. Statistical Analysis

Results are expressed as mean values ± S.E.M. Kruskal-Wallis followed by Dunn tests were used to compare Control, Pregestational and Gestational groups using Prism software (GraphPad, San Diego, CA, USA). When comparing only two groups a Mann-Whitney test was performed. Values of *p* < 0.05 were considered statistically significant. 

## 3. Results

One week after the last immunization with *T. gondii* lysate, the anti-*T.gondii* IgG seropositivity was evaluated in the serum samples of female rats from the control and the Pregestational groups. As was expected, anti-*T gondii* IgG seropositivity was found in the Pregestational group of immunized mothers (OD: 2.66 ± 0.25), while in the control and Gestational groups showed no reactivity (OD: 1.58 ± 0.13). Once anti-*T. gondii* IgG seropositivity was confirmed, the female rats were mated. Locomotor, cognitive and social behaviors, as well as cortical neurotransmitters, were evaluated in the progeny of the three experimental groups at postnatal day 60 (PND 60). It should be noted that no changes in body or brain weight were found among the progeny of the experimental groups compared to the control group.

### 3.1. Pregestational T. gondii Lysate Administration Induces Locomotor Alterations in the Progeny

**Figure 1:**
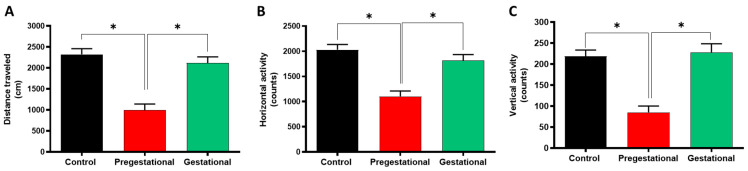
Effect of maternal immunization with *T. gondii* lysate on the (**A**) distance traveled, (**B**) horizontal activity and (**C**) vertical activity of the progeny. The locomotor activity was evaluated at PND 60 of the progeny of mothers that received PBS (control), the progeny of mothers immunized previously to gestation (Pregestational) and the progeny of mothers injected with *T. gondii* lysate at gestational day 18 (Gestational). Data are the mean ± SEM (n = 12–15 per group). * *p* < 0.01, based on the Kruskal–Wallis test with Dunn’s test for multiple pairwise comparisons.

Locomotor activity was assessed through three parameters: distance traveled, and horizontal and vertical movements (Figure 1A–C, respectively). The progeny of mothers immunized before gestation (Pregestational) with *T. gondii* lysate travelled 57.5 ± 7.1% less distance than the progeny from the control group (mothers that received three doses of subcutaneous PBS before gestation). A similar effect was observed for the other two parameters, the Pregestational group showed a decrease of 45.7 ± 5.2% in horizontal movements, and 61.0 ± 6.9% in vertical movements compared to the control group. In contrast, the progeny of mothers administrated with *T. gondii* lysate on gestational day 18 (Gestational) showed no changes in any of the parameters evaluated compared to the control. 

### 3.2. Cognitive Alteration Induced by the Maternal Immunization with T. gondii Lysate

**Figure 2:**
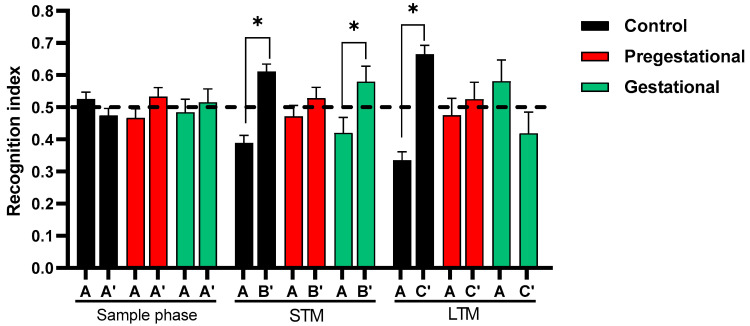
Evaluation of familiarization, short-term memory (STM) and long-term memory (LTM) phases in the progeny of rats exposed to *T. gondii* lysate before gestation (Pregestational) and on day 18 of gestation (Gestational) through NOR. The data represent mean ± S.E.M. recognition index (time exploring novel object/time exploring both objects × 100) of 12–15 animals per group. * *p* < 0.05 between the novel and familiar objects. Mann-Whitney U test.

Cognitive changes in the progeny induced by the maternal immunization with *T. gondii* lysate were evaluated in the NOR test in three phases: (1) sample phase, in which two identical objects (A and A’) are placed in the cage and the rats are free to examine the objects; (2) short-term memory evaluation, in which one of the familiar objects used on the previous phase is changed for a new one B; and (3) long term memory evaluation, in which the object B is changed by a new object C. The objects used in this test are of the same size and texture, they only vary in shape and color. The recognition index obtained for each phase and each experimental group represents the difference in time that the animal spent examining the new object in comparison to the known object. In the sample phase, all groups were able to identify objects A and A’ in a similar manner, since the recognition index obtained for the control, Pregestational and Gestational groups identified object A and A’ similarly. As expected, the control group spent more time with the novel object B when short memory was evaluated, this effect also being observed in the Gestational group. However, the Pregestational group was unable to discriminate between the familiar object and the new object indicating impairment in short-term memory. When long-term memory was evaluated control group spent more time with the new object. However, neither the Pregestational group, nor the Gestational group was able to discriminate the novel object (C) from the familiar one (A), indicating a deficit in long-term memory (Figure 2).

### 3.3. Social Behavior Alteration on the Progeny of Mothers Exposed to T. gondii Lysate

**Figure 3:**
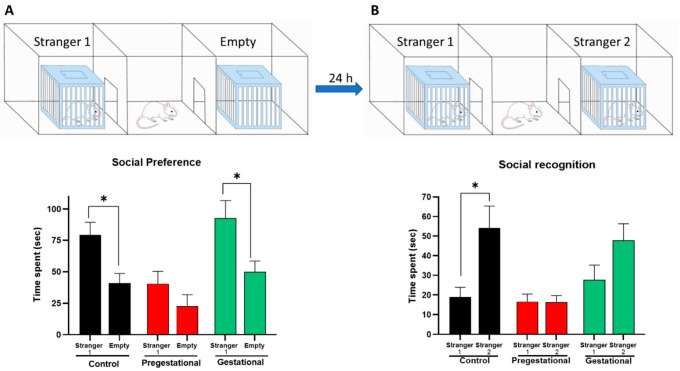
Effect of maternal immunization with a *T. gondii* lysate on social interaction of the offspring. At PND 60, social preference (**A**) and social recognition (**B**) were evaluated through the Crawley test in the progeny of mothers immunized before pregnancy (Pregestational) and mothers immunized on day 18 of gestation (Gestational). Data represent mean ± S.E.M. of time spent with the different stimuli of 12–15 animals per group. * *p* < 0.05 Mann-Whitney U test.

Social interaction was assessed using the Crawley test (Figure 3). In the first phase of the test, it was observed that the progeny belonging to the control group spent more time exploring the unknown rodent (stranger 1) than the empty chamber; this same behavior occurred in the Gestational group (Figure 3A). However, the Pregestational group did not show any preference for exploring the unknown rodent over the empty chamber, and in fact the total exploration time was reduced (~50%) compared to the control group and Gestational groups, which is consistent with the decreased locomotor activity shown by these animals, i.e., this group travels half the distance of the control and Gestational groups. 

In the second stage of this test social recognition and consequently attraction to novelty (stranger 2) was evaluated. As expected, the control group spent a longer time exploring the unknown rat than the familiar. Moreover, the Gestational group tended to explore the unknown rodent more than the known one, but this difference was not significant. Interestingly, the Pregestational group was unable to discriminate the known rat from the unknown one and continued showing disinterest in interacting with any of them, indicating a decrease of social behavior (Figure 3B).

### 3.4. Neurochemical Alterations on the Progeny of Mothers Exposed to T. gondii Lysate

**Figure 4:**
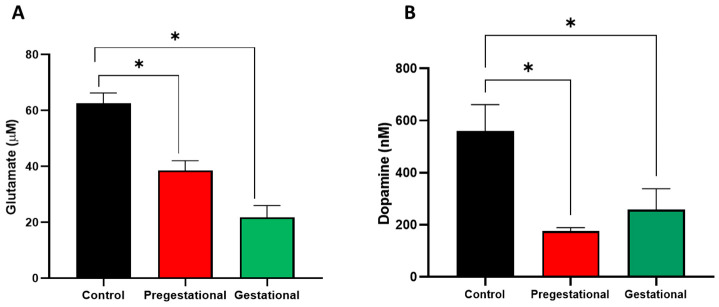
Effect of maternal exposure of *T. gondii* lysate on cortical neurotransmitters levels in offspring. The cortical levels of (**A**) glutamate and (**B**) dopamine were evaluated in the three experimental groups at PND 60 (PBS: control, Pregestational: the progeny of females immunized before pregnancy and Gestational: the progeny of mothers exposed to *T. gondii* lysate on gestational day 18). Data represent mean ± S.E.M. of 12–15 animals per group. Kruskal-Wallis followed by Dunn’s test, * *p* < 0.01.

After behavioral tests, brain tissue was obtained, and glutamate and dopamine were determined in the brain cortex of experimental groups. As seen in Figure 4A, cortical glutamate levels are significantly decreased in the Pregestational and Gestational groups compared to the control (38.4% and 65.3% vs. control, respectively). Similarly, cortical dopamine levels decreased by 68.6% and 53.8% for Pregestational and Gestational groups, respectively, compared to the control group.

### 3.5. Formation of Pathogenic Antibodies Directed against Fetal Brain Proteins

**Figure 5:**
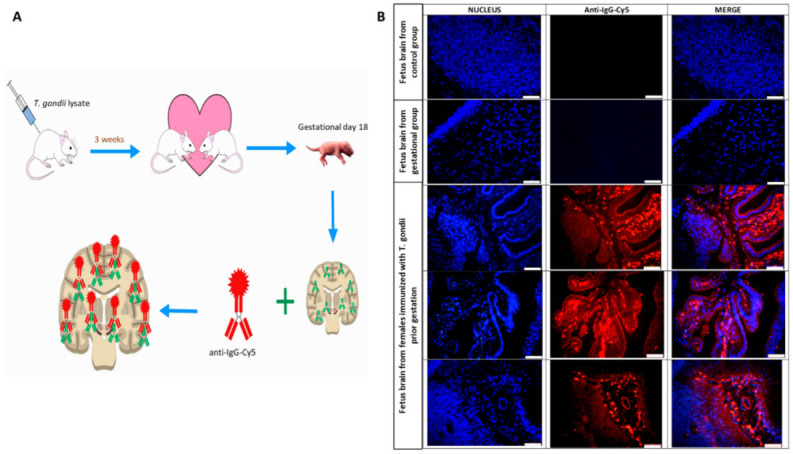
Anti-*T. gondii* IgG attached to fetal brain structures. (**A**) Diagram of the experimental design. Rats immunized prior to gestation or control, received one subcutaneous injection with *T. gondii* lysate or PBS, respectively, before being pregnant, for three weeks. Fetal brains from the experimental groups obtained at gestational day 18 were used to search IgG bound to brain structures. (**B**) Representative brain slice images of fetuses from the experimental groups on gestational day 18. IgGs bound to brain structures were revealed using an anti-IgG-Cy5 antibody (red). Nuclei were counterstained with DAPI (blue). Images were acquired at 40×, the bar representing 100 µm.

Once the behavioral and neurochemical alterations were evidenced in the Pregestational group, two strategies were followed to demonstrate whether these alterations were due to *T. gondii* sharing similar epitopes with immature brain proteins, which may result in structural and functional impairments in the offspring. The first strategy consisted in identify IgGs bound to the immature brain (gestational day 18) of the progeny of mothers immunized with the *T. gondii* lysate prior to pregnancy (Pregestational group, Figure 5A). As can be seen in Figure 5B, the immature brains of control group (mothers injected with PBS) as well as the Gestational group showed no reactivity when incubated with the Cy5-coupled anti-rat IgG antibody (fluorescence in red). However, the immature offspring brains from rats immunized before pregnancy showed strong reactivity when incubated with the same secondary anti-IgG-Cy5 antibody, indicating the presence of IgGs bound to various brain regions.

**Figure 6:**
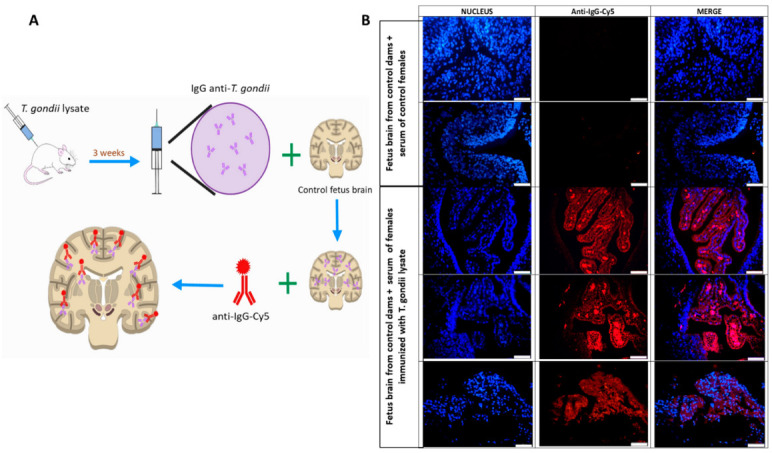
Maternal pathogenic antibodies recognized fetal brain structures. (**A**) Diagram of the experimental design. Rats immunized with the *T. gondii* lysate or control, received three subcutaneous injections (one per week) with *T. gondii* lysate or PBS, respectively. Fetus brains at gestational day 18 from the progeny of a non-immunized mother were used to search cross-reactive IgGs. (**B**) Representative images of brain slices of fetuses on gestational day 18 from non-immunized mothers were incubated with serum from females previously immunized with *T. gondii*. The binding of IgG’s was subsequently evidenced by a secondary anti-rat-IgG-Cy5 (red). Nuclei were counterstained with DAPI (blue). Images were acquired at 40×, the bar represents 100 µm.

Moreover, the second strategy to demonstrate the possible molecular mimicry between *T. gondii* and neurodevelopmental proteins consisted in incubating the immature brain of the progeny of control rats with the serum of female rats immunized with the *T. gondii* lysate. Therefore, if *T. gondii* proteins have structural similarity with neurodevelopmental proteins, maternal IgGs from the serum of immunized mothers would cross-react with antigens of the immature brain (Figure 6A). As seen in Figure 6B, when a control immature brain is incubated with serum from a PBS-injected rats there is no positive reactivity for the anti-IgG-Cy5. In contrast, when the immature brain is exposed to the maternal serum of rats immunized with *T. gondii* lysate (serum with anti-*T. gondii* IgG), the binding of these IgGs to various fetal brain structures was evidenced by the fluorescence marker anti-IgG-Cy5 antibody; a wide distribution of IgG signal within the immature brain is shown in Figure 6B.

Taken together, these data demonstrate that it is plausible that *T. gondii* shares antigenic determinants with immature brain structures which induce the formation of pathogenic anti-*T. gondii* IgGs that recognize fetal brain structures as *T. gondii* proteins.

**Figure 7:**
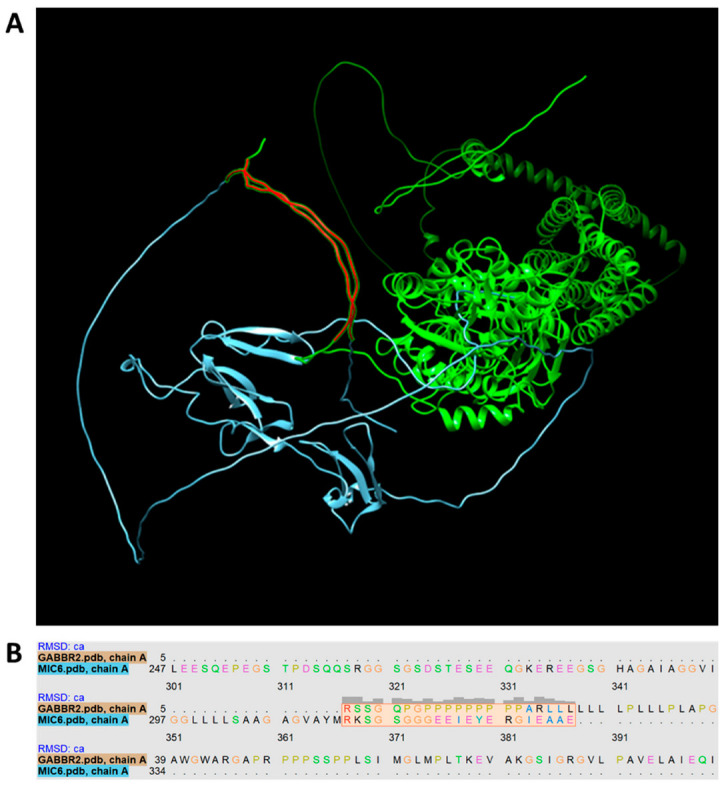
Structural comparison between GABBR2 central nervous system protein (green) and MIC6 *T. gondii* protein (cyan). (**A**) The three-dimensional structures of the GABBR2 and MIC6 proteins were downloaded from the Uniprot platform and aligned using the UCSF Chimera program, shared sequences between both proteins in red. (**B**) The Matchmaker extension performs the alignment of the sequences of both proteins and calculates the best alignment score. Amino acids 5 to 25 of GABBR2 and 314 to 334 of MIC6 have the best matching sequence.

After corroborating the occurrence of pathogenic maternal antibodies following *T. gondii* lysate immunization, the next step was to explore the possibility of shared mimotopes between *T. gondii* proteins and brain proteins. To identify possible similarities, we performed a first exploratory analysis using the three-dimensional structures available in the UCSF Chimera program. The three-dimensional models of the brain protein Gamma-Aminobutyric Acid Type B Receptor (GABBR2) and the T. gondii integral membrane protein MIC6, that plays an essential role in host cell attachment, were compared and aligned structurally (Figure 7A). Next, once a structural similarity was found we used the Matchmaker extension to retrieve the alignment of the aminoacidic sequences of both proteins; the region composed of the amino acids 5 to 25 of the GABBR2 protein conforms to a structure similar to the segment formed by amino acids 314 to 334 of the MIC6 protein as shown in the pairwise aminoacidic alignment (Figure 7B). 

## 4. Discussion

Epidemiological studies have shown that the activation of the maternal immune system caused by infections increases the risk of developing schizophrenia and other psychiatric disorders in adult offspring [9,11,28,29,30]. Particularly, maternal exposure to *T. gondii* is associated with schizophrenia in the offspring [10,20]. Furthermore, it was demonstrated that the high titers of anti-*T. gondii* IgG found in the dried neonatal blood samples from a biobank were associated with an increased risk of schizophrenia [31]. These data on the relationship between maternal *T. gondii* infection and the risk of schizophrenia in the offspring suggested that IgG antibodies cross the placenta during pregnancy and are transmitted to the fetus [32]. Here, we confirm the gestational transmission of maternal anti-*T. gondii* IgG to the offspring, and for the first time we demonstrated that these maternal antibodies bind to the immature brain by recognizing fetal brain mimotopes. The binding of these pathogenic antibodies to the developing brain provides a possible explanation of causality for the neurochemical, cognitive and social alterations later observed in the offspring of mothers immunized with *T. gondii* lysate before gestation. 

Psychiatric disorders related to the maternal *T. gondii* infection had been associated with the vertical transfer from the mother to the fetus, without considering long-term immune maternal alterations previously induced by the presence of this parasite. In this study, we used a *T. gondii* lysate to avoid the cognitive alterations induced by the presence of the pathogen in the CNS, which might complicate data interpretation. Herein, two experimental models of exposure to *T. gondii* were performed: (1) Pregestational model—females were immunized with *T. gondii* lysate before gestation, in which the anti-*T. gondii* IgG seropositivity was corroborated; and (2) Gestational model—mothers were administrated with *T. gondii* lysate at gestational day 18, which represents an acute MIA [33]. Both experimental models showed neurochemical and cognitive alterations but with different profiles and magnitudes. In this line, several studies have shown that the MIA with LPS or polyriboinosinic polyribocytidylic acid (Poly(I:C)) induces alterations in exploratory behavior, social interaction, cognitive function and sensorimotor gating in adult offspring [15,34,35]. However, these models do not consider the pathogen-specific adaptative cellular immune response induced by an infectious pathogen. Taking this into consideration, a recent study showed that the maternal immune induction by the exposure of *T. gondii* lysate on gestational day 14 produces behavioral abnormalities, brain microstructure alterations and a pro-inflammatory CD4 + T cell profile in the adult offspring [33]. Herein, our data confirmed that the MIA by *T. gondii lysate* induces neurochemical, cognitive and social alterations in the adult offspring, which could be attributed to the pro-inflammatory immune response induced by T-cells according to the previous evidence shown by Xu et al. [33].

On the other hand, the Pregestational model used in this study simulates *T. gondii* maternal infection prior to the gestational period with plenty of time for the antibodies previously produced against the parasite to be transferred to the fetus. During fetal neurodevelopment, the IgGs are permitted to cross the developing blood–brain barrier [36]. However, it has not previously been explored whether some of these maternal IgGs could present reactivity to fetal brain antigens. Supporting this idea, we demonstrated the direct interaction between maternal anti-*T. gondii* IgG and fetal brain mimotopes since IgGs were found to be bound to the fetal brain structures of the Pregestational group, contrasted with the progeny from mothers exposed to lysate during gestation (Gestational group), indicating that the neurochemical and cognitive alterations observed on the adult offspring are due to different mechanisms between these both groups. The findings on the Pregestational group confirmed that the neurochemical and cognitive alterations may be attributed to the pathogenic maternal cross-reactive IgGs which recognize fetal brain structures. A similar murine study using a single dose of brain-reactive IgG antibodies from mothers of children with autism to pregnant mice showed that the passive gestational transfer of IgG induced anxiety, delayed motor development, and lower social interaction on the progeny [23]. Based on these background and our results, it is suggested that the alterations observed in the progeny of the Pregestational group are derived from a process known as molecular mimicry, in which maternal anti-*T. gondii* IgGs recognize fetal brain antigens as *T. gondii* antigens. This phenomenon could be associated with the causality of psychiatric diseases even in the absence of an active infection. In addition, we explored the antigenic mimicry hypothesis comparing gamma-aminobutyric acid type B receptor subunit 2 (GABBR2), a brain protein essential for neurotransmission, with the amino acids sequence of a *T. gondii* protein. We showed that GABBR2 shared an amino acid sequence with the microneme protein 6 (MIC6) of *T. gondii*. MIC6 is a highly immunogenic protein responsible for parasite adhesion and invasion, and has been considered a potential vaccine candidate against toxoplasmosis [37,38]. At present, our group is focusing on the immunoinformatic research for potential mimotopes between CNS and *T. gondii* proteins, taking into account their antigenicity profiles.

It is feasible that early brain development disruption caused by maternal pathogenic antibodies or by proinflammatory cytokines could alter the trajectory of brain growth and function across the lifespan. In this study, we also observed a cortical decrease in dopamine and glutamate levels in the Pregestational and Gestational groups, which could be related to the hypofunction, cognitive impairment and disrupted social behavior observed in these experimental groups. In this line, it has been demonstrated that neurotransmission is disturbed in schizophrenia, especially in the dopamine, glutamate, GABA and serotonin systems [39,40]. Particularly, dopamine—a neurotransmitter involved in multiple functions including movement, cognition, and emotional regulations—is decreased in schizophrenic patients, who present negative symptoms and cognitive impairment [41,42]. In addition, glutamatergic transmission is also related to schizophrenia-negative symptoms, based on clinical observations in which NMDAr antagonists such as ketamine and phencyclidine induced cognitive impairments and physiological disturbances in normal subjects in a similar way to schizophrenic patients [43,44,45,46]. Thus, the alterations produced by molecular mimicry between *T. gondii* and fetal brain structures could reproduce the neurochemical abnormalities as well as abnormal connections leading to an ineffective communication between brain critical areas responsible for cognitive and psychosocial functions, such as those observed in this work. Moreover, brain dopamine and glutamate levels alterations induced by molecular mimicry could also be involved in the dysregulation of levels of kynurenic acid, a kynurenine pathway metabolite, able to modulate the glutamatergic, cholinergic, and dopaminergic transmission and the levels of which are increased in the CFS or tissue of schizophrenia patients [47,48,49].

Altogether these data demonstrate that the maternal pathogenic antibodies produced by *T. gondii* immunization recognize fetal brain mimotopes and induce changes in brain function reflected by neurochemical alterations as well as disrupted locomotor activity, cognitive abilities and social behavior similar to negative symptoms observed in schizophrenia, highlighting new directions for further investigations in which the prenatal diagnostic could reduce the risk to develop psychiatric disorders on the offspring. The challenge for future studies is to determine whether the alterations may be triggered earlier by an environmental stressor or if they can be prevented by blocking antibodies able to avoid the functional alterations that later develop in the adult offspring.

## 5. Conclusions

Taken together, these data demonstrate that *T. gondii* shares antigenic determinants with immature brain structures, thus leading to the formation of pathogenic anti-*T. gondii* that recognize fetal brain structures, which in turn lead to behavioral and memory impairments. What remains a major challenge is to dissect the specific fetal brain mimotopes that led to disrupting the neuronal circuits and to neurodevelopmental defects which later induce neurochemical, behavioral and social alterations. These future studies should consider the time in which the *T. gondii* infection occurs, since the spectrum of associated psychiatric disorders is different and is related to the kind of the induced immune response, whether innate or adaptive. Therefore, future research needs to identify target brain protein mimotopes and their connection to neuronal circuits in the healthy and diseased brain, hopefully elucidating the causality, prevention or treatment of schizophrenia is feasible.

## Data Availability

The data presented in this study are available on request from the corresponding author.

## References

[1] Matta S.K., Rinkenberger N., Dunay I.R., Sibley L.D. (2021). Toxoplasma gondii infection and its implications within the central nervous system. Nat. Rev. Microbiol..

[2] Fišar Z. (2022). Biological hypotheses, risk factors, and biomarkers of schizophrenia. Prog. Neuro-Psychopharmacol. Biol. Psychiatry.

[3] Piper M., Beneyto M., Burne T.H., Eyles D.W., Lewis D.A., McGrath J.J. (2012). The neurodevelopmental hypothesis of schizophrenia: Convergent clues from epidemiology and neuropathology. Psychiatr. Clin. N. Am..

[4] Reichard J., Zimmer-Bensch G. (2021). The Epigenome in Neurodevelopmental Disorders. Front. Neurosci..

[5] Pantelis C., Velakoulis D., McGorry P.D., Wood S.J., Suckling J., Phillips L.J., Yung A.R., Bullmore E.T., Brewer W., Soulsby B. (2003). Neuroanatomical abnormalities before and after onset of psychosis: A cross-sectional and longitudinal MRI comparison. Lancet.

[6] Pantelis C., Yucel M., Wood S.J., Velakoulis D., Sun D., Berger G., Stuart G.W., Yung A., Phillips L., McGorry P.D. (2005). Structural brain imaging evidence for multiple pathological processes at different stages of brain development in schizophrenia. Schizophr. Bull..

[7] Selemon L.D., Goldman-Rakic P.S. (1999). The reduced neuropil hypothesis: A circuit based model of schizophrenia. Biol. Psychiatry.

[8] Brown A.S., Begg M.D., Gravenstein S., Schaefer C.A., Wyatt R.J., Bresnahan M., Babulas V.P., Susser E.S. (2004). Serologic evidence of prenatal influenza in the etiology of schizophrenia. Arch. Gen. Psychiatry.

[9] Brown A.S., Cohen P., Greenwald S., Susser E. (2000). Nonaffective psychosis after prenatal exposure to rubella. Am. J. Psychiatry.

[10] Brown A.S., Schaefer C.A., Quesenberry C.P., Liu L., Babulas V.P., Susser E.S. (2005). Maternal exposure to toxoplasmosis and risk of schizophrenia in adult offspring. Am. J. Psychiatry.

[11] Buka S.L., Tsuang M.T., Torrey E.F., Klebanoff M.A., Bernstein D., Yolken R.H. (2001). Maternal infections and subsequent psychosis among offspring. Arch. Gen. Psychiatry.

[12] Patterson P.H. (2009). Immune involvement in schizophrenia and autism: Etiology, pathology and animal models. Behav. Brain Res..

[13] Urakubo A., Jarskog L.F., Lieberman J.A., Gilmore J.H. (2001). Prenatal exposure to maternal infection alters cytokine expression in the placenta, amniotic fluid, and fetal brain. Schizophr. Res..

[14] Samuelsson A.M., Jennische E., Hansson H.A., Holmang A. (2006). Prenatal exposure to interleukin-6 results in inflammatory neurodegeneration in hippocampus with NMDA/GABA(A) dysregulation and impaired spatial learning. Am. J. Physiol. Regul. Integr. Comp. Physiol..

[15] Cai Z., Pan Z.L., Pang Y., Evans O.B., Rhodes P.G. (2000). Cytokine induction in fetal rat brains and brain injury in neonatal rats after maternal lipopolysaccharide administration. Pediatr. Res..

[16] Jonakait G.M. (2007). The effects of maternal inflammation on neuronal development: Possible mechanisms. Int. J. Dev. Neurosci..

[17] Brown A.S., Susser E.S. (2002). In utero infection and adult schizophrenia. Ment. Retard. Dev. Disabil. Res. Rev..

[18] Dammann O., Leviton A. (1997). Maternal intrauterine infection, cytokines, and brain damage in the preterm newborn. Pediatr. Res..

[19] Gilmore J.H., Fredrik Jarskog L., Vadlamudi S., Lauder J.M. (2004). Prenatal infection and risk for schizophrenia: IL-1beta, IL-6, and TNFalpha inhibit cortical neuron dendrite development. Neuropsychopharmacology.

[20] Mortensen P.B., Norgaard-Pedersen B., Waltoft B.L., Sorensen T.L., Hougaard D., Yolken R.H. (2007). Early infections of Toxoplasma gondii and the later development of schizophrenia. Schizophr. Bull..

[21] Martin L.A., Ashwood P., Braunschweig D., Cabanlit M., Van de Water J., Amaral D.G. (2008). Stereotypies and hyperactivity in rhesus monkeys exposed to IgG from mothers of children with autism. Brain Behav. Immun..

[22] Singer H.S., Morris C., Gause C., Pollard M., Zimmerman A.W., Pletnikov M. (2009). Prenatal exposure to antibodies from mothers of children with autism produces neurobehavioral alterations: A pregnant dam mouse model. J. Neuroimmunol..

[23] Braunschweig D., Golub M.S., Koenig C.M., Qi L., Pessah I.N., Van de Water J., Berman R.F. (2012). Maternal autism-associated IgG antibodies delay development and produce anxiety in a mouse gestational transfer model. J. Neuroimmunol..

[24] Lee J.Y., Huerta P.T., Zhang J., Kowal C., Bertini E., Volpe B.T., Diamond B. (2009). Neurotoxic autoantibodies mediate congenital cortical impairment of offspring in maternal lupus. Nat. Med..

[25] Sonaimuthu P., Ching X.T., Fong M.Y., Kalyanasundaram R., Lau Y.L. (2016). Induction of Protective Immunity against Toxoplasmosis in BALB/c Mice Vaccinated with Toxoplasma gondii Rhoptry-1. Front. Microbiol..

[26] Tyebji S., Seizova S., Garnham A.L., Hannan A.J., Tonkin C.J. (2019). Impaired social behaviour and molecular mediators of associated neural circuits during chronic Toxoplasma gondii infection in female mice. Brain Behav. Immun..

[27] Kaidanovich-Beilin O., Lipina T., Vukobradovic I., Roder J., Woodgett J.R. (2011). Assessment of social interaction behaviors. J. Vis. Exp..

[28] Mednick S.A., Machon R.A., Huttunen M.O., Bonett D. (1988). Adult schizophrenia following prenatal exposure to an influenza epidemic. Arch. Gen. Psychiatry.

[29] Brown A.S. (2006). Prenatal infection as a risk factor for schizophrenia. Schizophr. Bull..

[30] Brown A.S. (2012). Epidemiologic studies of exposure to prenatal infection and risk of schizophrenia and autism. Dev. Neurobiol..

[31] Mortensen P.B., Norgaard-Pedersen B., Waltoft B.L., Sorensen T.L., Hougaard D., Torrey E.F., Yolken R.H. (2007). Toxoplasma gondii as a risk factor for early-onset schizophrenia: Analysis of filter paper blood samples obtained at birth. Biol. Psychiatry.

[32] Malek A., Sager R., Kuhn P., Nicolaides K.H., Schneider H. (1996). Evolution of maternofetal transport of immunoglobulins during human pregnancy. Am. J. Reprod. Immunol..

[33] Xu Z., Zhang X., Chang H., Kong Y., Ni Y., Liu R., Zhang X., Hu Y., Yang Z., Hou M. (2021). Rescue of maternal immune activation-induced behavioral abnormalities in adult mouse offspring by pathogen-activated maternal Treg cells. Nat. Neurosci..

[34] Meyer U., Feldon J. (2012). To poly(I:C) or not to poly(I:C): Advancing preclinical schizophrenia research through the use of prenatal immune activation models. Neuropharmacology.

[35] Shi L., Smith S.E., Malkova N., Tse D., Su Y., Patterson P.H. (2009). Activation of the maternal immune system alters cerebellar development in the offspring. Brain Behav. Immun..

[36] Bake S., Friedman J.A., Sohrabji F. (2009). Reproductive age-related changes in the blood brain barrier: Expression of IgG and tight junction proteins. Microvasc. Res..

[37] Peng G.H., Yuan Z.G., Zhou D.H., He X.H., Liu M.M., Yan C., Yin C.C., He Y., Lin R.Q., Zhu X.Q. (2009). Toxoplasma gondii microneme protein 6 (MIC6) is a potential vaccine candidate against toxoplasmosis in mice. Vaccine.

[38] Xu X.P., Liu W.G., Xu Q.M., Zhu X.Q., Chen J. (2019). Evaluation of immune protection against Toxoplasma gondii infection in mice induced by a multi-antigenic DNA vaccine containing TgGRA24, TgGRA25 and TgMIC6. Parasite.

[39] Coyle J.T., Konopaske G.T., Brady S.T., Siegel G.J., Albers R.W., Price D.L. (2012). Chapter 58-The Neurochemistry of Schizophrenia. Basic Neurochemistry.

[40] Anderson G., Maes M. (2013). Schizophrenia: Linking prenatal infection to cytokines, the tryptophan catabolite (TRYCAT) pathway, NMDA receptor hypofunction, neurodevelopment and neuroprogression. Prog. Neuropsychopharmacol. Biol. Psychiatry.

[41] Konopaske G.T., Coyle J.T., Zigmond M.J., Wiley C.A., Chesselet M.-F. (2023). Chapter 46-The neurobiology of schizophrenia∗. Neurobiology of Brain Disorders.

[42] Bressan R.A., Crippa J.A. (2005). The role of dopamine in reward and pleasure behaviour--review of data from preclinical research. Acta Psychiatr. Scand. Suppl..

[43] Luby E.D., Cohen B.D., Rosenbaum G., Gottlieb J.S., Kelley R. (1959). Study of a new schizophrenomimetic drug; sernyl. AMA Arch. Neurol. Psychiatry.

[44] Anis N.A., Berry S.C., Burton N.R., Lodge D. (1983). The dissociative anaesthetics, ketamine and phencyclidine, selectively reduce excitation of central mammalian neurones by *N*-methyl-aspartate. Br. J. Pharmacol..

[45] Coyle J.T. (2006). Glutamate and schizophrenia: Beyond the dopamine hypothesis. Cell. Mol. Neurobiol..

[46] Coyle J.T., Basu A., Benneyworth M., Balu D., Konopaske G. (2012). Glutamatergic synaptic dysregulation in schizophrenia: Therapeutic implications. Handb. Exp. Pharmacol..

[47] Schwarcz R., Hunter C.A. (2007). Toxoplasma gondii and schizophrenia: Linkage through astrocyte-derived kynurenic acid?. Schizophr. Bull..

[48] Schwarcz R., Rassoulpour A., Wu H.Q., Medoff D., Tamminga C.A., Roberts R.C. (2001). Increased cortical kynurenate content in schizophrenia. Biol. Psychiatry.

[49] Sathyasaikumar K.V., Stachowski E.K., Wonodi I., Roberts R.C., Rassoulpour A., McMahon R.P., Schwarcz R. (2011). Impaired kynurenine pathway metabolism in the prefrontal cortex of individuals with schizophrenia. Schizophr. Bull..

